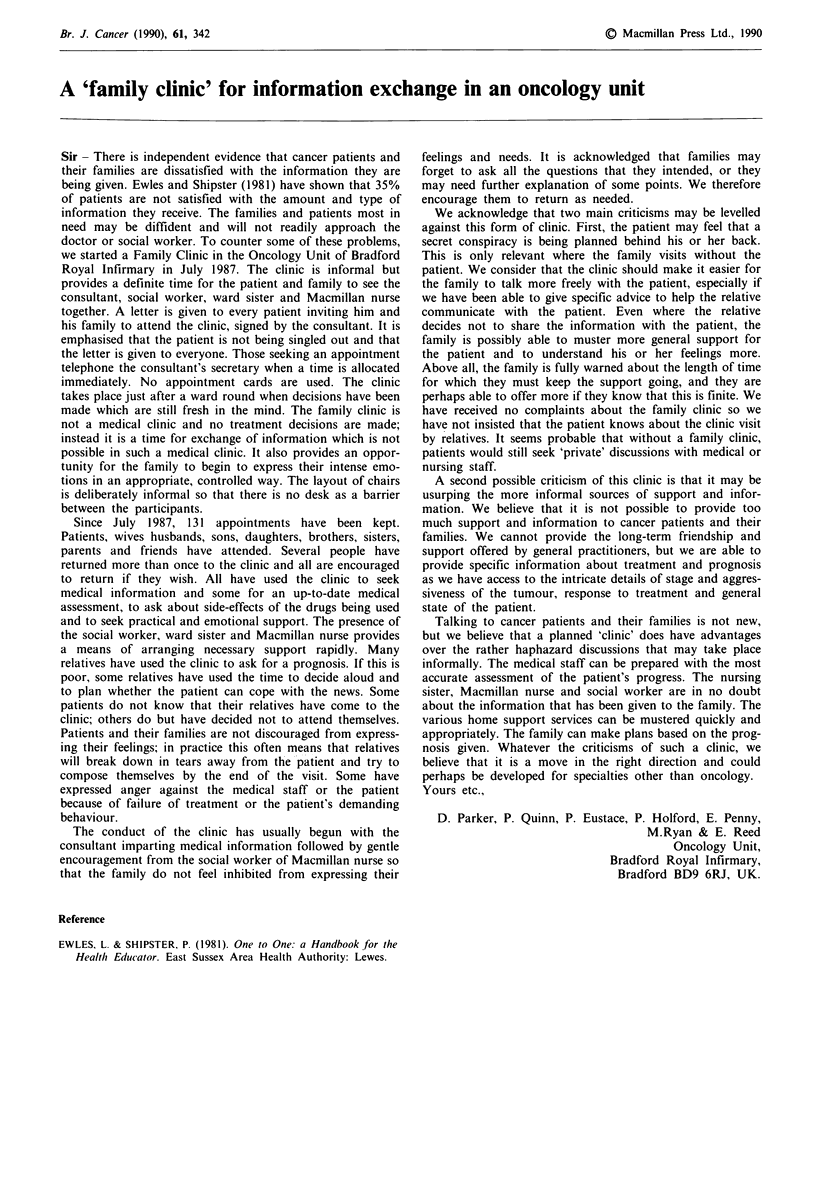# A 'family clinic' for information exchange in an oncology unit.

**DOI:** 10.1038/bjc.1990.71

**Published:** 1990-02

**Authors:** D. Parker, P. Quinn, P. Eustace, P. Holford, E. Penny, M. Ryan, E. Reed


 © Macmillan Press Ltd., 1990

### A 'family clinic' for information exchange in an oncology unit

**Sir** – There is independent evidence that cancer patients and their families are dissatisfied with the information they are being given. Ewles and Shipster (1981) have shown that 35% of patients are not satisfied with the amount and type of information they receive. The families and patients most in need may be diffident and will not readily approach the doctor or social worker. To counter some of these problems, we started a Family Clinic in the Oncology Unit of Bradford Royal Infirmary in July 1987. The clinic is informal but provides a definite time for the patient and family to see the consultant, social worker, ward sister and Macmillan nurse together. A letter is given to every patient inviting him and his family to attend the clinic, signed by the consultant. It is emphasised that the patient is not being singled out and that the letter is given to everyone. Those seeking an appointment telephone the consultant's secretary when a time is allocated immediately. No appointment cards are used. The clinic takes place just after a ward round when decisions have been made which are still fresh in the mind. The family clinic is not a medical clinic and no treatment decisions are made; instead it is a time for exchange of information which is not possible in such a medical clinic. It also provides an opportunity for the family to begin to express their intense emotions in an appropriate, controlled way. The layout of chairs is deliberately informal so that there is no desk as a barrier between the participants.

Since July 1987, 131 appointments have been kept. Patients, wives husbands, sons, daughters, brothers, sisters, parents and friends have attended. Several people have returned more than once to the clinic and all are encouraged to return if they wish. All have used the clinic to seek medical information and some for an up-to-date medical assessment, to ask about side-effects of the drugs being used and to seek practical and emotional support. The presence of the social worker, ward sister and Macmillan nurse provides a means of arranging necessary support rapidly. Many relatives have used the clinic to ask for a prognosis. If this is poor, some relatives have used the time to decide aloud and to plan whether the patient can cope with the news. Some patients do not know that their relatives have come to the clinic; others do but have decided not to attend themselves. Patients and their families are not discouraged from expressing their feelings; in practice this often means that relatives will break down in tears away from the patient and try to compose themselves by the end of the visit. Some have expressed anger against the medical staff or the patient because of failure of treatment or the patient's demanding behaviour.

The conduct of the clinic has usually begun with the consultant imparting medical information followed by gentle encouragement from the social worker of Macmillan nurse so that the family do not feel inhibited from expressing their feelings and needs. It is acknowledged that families may forget to ask all the questions that they intended, or they may need further explanation of some points. We therefore encourage them to return as needed.

We acknowledge that two main criticisms may be levelled against this form of clinic. First, the patient may feel that a secret conspiracy is being planned behind his or her back. This is only relevant where the family visits without the patient. We consider that the clinic should make it easier for the family to talk more freely with the patient, especially if we have been able to give specific advice to help the relative communicate with the patient. Even where the relative decides not to share the information with the patient, the family is possibly able to muster more general support for the patient and to understand his or her feelings more. Above all, the family is fully warned about the length of time for which they must keep the support going, and they are perhaps able to offer more if they know that this is finite. We have received no complaints about the family clinic so we have not insisted that the patient knows about the clinic visit by relatives. It seems probable that without a family clinic, patients would still seek 'private' discussions with medical or nursing staff.

A second possible criticism of this clinic is that it may be usurping the more informal sources of support and information. We believe that it is not possible to provide too much support and information to cancer patients and their families. We cannot provide the long-term friendship and support offered by general practitioners, but we are able to provide specific information about treatment and prognosis as we have access to the intricate details of stage and aggressiveness of the tumour, response to treatment and general state of the patient.

Talking to cancer patients and their families is not new, but we believe that a planned 'clinic' does have advantages over the rather haphazard discussions that may take place informally. The medical staff can be prepared with the most accurate assessment of the patient's progress. The nursing sister, Macmillan nurse and social worker are in no doubt about the information that has been given to the family. The various home support services can be mustered quickly and appropriately. The family can make plans based on the prognosis given. Whatever the criticisms of such a clinic, we believe that it is a move in the right direction and could perhaps be developed for specialties other than oncology.

Yours etc.,

D. Parker, P. Quinn, P. Eustace, P. Holford, E. Penny, M.Ryan & E. Reed
Oncology Unit,
Bradford Royal Infirmary,
Bradford BD9 6RJ, UK.

**Reference**

EWLES, L. & SHIPSTER, P. (1981). *One to One: a Handbook for the Health Educator.* East Sussex Area Health Authority: Lewes.